# Brain targeted oral delivery of doxycycline hydrochloride encapsulated Tween 80 coated chitosan nanoparticles against ketamine induced psychosis: behavioral, biochemical, neurochemical and histological alterations in mice

**DOI:** 10.1080/10717544.2017.1377315

**Published:** 2017-09-25

**Authors:** Monu Yadav, Milind Parle, Nidhi Sharma, Sameer Dhingra, Neha Raina, Deepak Kumar Jindal

**Affiliations:** a Faculty of Medical Sciences, Department of Pharmaceutical Sciences, Guru Jambheshwar University of Science & Technology, Hisar, India;; b Faculty of Medical Sciences, School of Pharmacy, The University of the West Indies, St. Augustine, Trinidad and Tobago

**Keywords:** Doxycycline hydrochloride, chitosan, nanoparticles, psychosis, ketamine

## Abstract

To develop statistically optimized brain targeted Tween 80 coated chitosan nanoparticulate formulation for oral delivery of doxycycline hydrochloride for the treatment of psychosis and to evaluate its protective effect on ketamine induced behavioral, biochemical, neurochemical and histological alterations in mice. 3^2^ full factorial design was used to optimize the nanoparticulate formulation to minimize particle size and maximize entrapment efficiency, while independent variables chosen were concentration of chitosan and Tween 80. The optimized formulation was characterized by particle size, drug entrapment efficiency, Fourier transform infrared, Transmission electron microscopy analysis and drug release behavior. Pure doxycycline hydrochloride (25 and 50 mg/kg, p.o.) and optimized doxycycline hydrochloride encapsulated Tween 80 coated chitosan nanoparticles (DCNP_opt_) (equivalent to 25 mg/kg doxycycline hydrochloride, p.o.) were explored against ketamine induced psychosis in mice. The experimental studies for DCNP_opt_, with mean particle size 237 nm and entrapment efficiency 78.16%, elucidated that the formulation successfully passed through blood brain barrier and exhibited significant antipsychotic activity. The underlying mechanism of action was further confirmed by behavioral, biochemical, neurochemical estimations and histopathological study. Significantly enhanced GABA and GSH level and diminished MDA, TNF-α and dopamine levels were observed after administration of DCNP_opt_ at just half the dose of pure doxycycline hydrochloride, showing better penetration of doxycyline hydrochloride in the form of Tween 80 coated nanoparticles through blood brain barrier. This study demonstrates the hydrophilic drug doxycycline hydrochloride, loaded in Tween 80 coated chitosan nanoparticles, can be effectively brain targeted through oral delivery and therefore represents a suitable approach for the treatment of psychotic symptoms.

## Introduction

Psychosis, a debilitating neuropsychiatric disorder, is characterized by positive, negative and cognitive symptoms. Complex pathophysiological mechanisms evolved in the progression of psychosis include GABAergic, dopaminergic, cholinergic dysfunction, hypofunctioning of NMDA receptors along with neuroinflammation and oxidative stress impedes its treatment (Kumar et al., 2017). Numerous drugs are available for the treatment of psychosis, but they provide only symptomatic relief (Rao & Remington, 2013; Hunter, 2014). Therefore, it is necessary to explore the drugs of different category to overcome this problem. In line with previous studies, tetracycline antibiotics possess neuroprotective, antidepressant, antianxiety, antipsychotics, memory enhancing, antioxidant and anti-inflammatory effects (Pae et al., 2008; Arakawa et al., 2012; Nagpal et al., 2013), thus, we have selected another tetracycline antibiotic, doxycycline, for the present study. Doxycycline hydrochloride has been used for its neuroprotective effect in previous study (Cho et al., 2009), as well as peripheral side effects appear at higher doses limiting its use for psychiatric disorders (Angelakis et al., 2015; Frye et al., 2015). Consequently, targeting doxycycline hydrochloride to brain can reduce its dose and overcome peripheral side effects for the treatment of psychiatric disorders, but its hydrophilicity deters it crossing the blood brain barrier. Nanoparticulate drug delivery systems have been designed for the targeting of drugs to brain for better efficacy (Kreuter, 2001, 2002). Hydrophilic drugs have been encapsulated in nanoparticles as carriers to improve their penetration in brain through blood brain barrier, enhance their bioavailability, minimize side effects as well as facilitate the controlled release of encapsulated drug (Hansraj et al., 2015). Enhanced permeability of Tween 80 coated chitosan nanoparticulate formulation has improved the penetration of drugs through blood brain barrier (Nagpal et al., 2013). In the present study, chitosan, a biopolymer, is used for the formulation of nanoparticles owing to its admirable properties like nontoxicity, biodegradability, biocompatibility and affordability, which has already been used to augment the penetration of drugs in the brain for the management of various psychiatric disorders (Fazil et al., 2012; Elnaggar et al., 2015; Guo, et al., 2016). Further, oral administration of Tween 80 coated chitosan nanoparticles provides protection against first pass effect and help to enter the systemic circulation which reach into brain via absorptive transcytosis besides minimizing peripheral side effects (Nagpal et al., 2013; Sarvaiya & Agrawal, 2015).

The objective of the present study was to develop an optimized formulation using 3^2^ full factorial design for brain targeting of doxycycline hydrochloride encapsulated Tween 80 coated chitosan nanoparticles, followed by evaluation of their antipsychotic activity in experimental model of ketamine induced psychosis.

## Materials and methods

### Materials

Doxycycline hydrochloride was purchased from Central Drug House (CDH) Laboratories Private Limited, India. Chitosan and sodium tripolyphosphate (TPP) were purchased from Hi-Media, Mumbai, India. Olanzapine and ketamine were obtained from Ranbaxy Laboratories Limited and Neon Pharmaceutical Private Limited, India, respectively. Other chemicals used in the present study were of suitable analytical reagent grade.

### Animals

Swiss albino male mice (3–4 month old; 25–30 g) were purchased from Disease Free Small Animal House, Lala Lajpat Rai University of Veterinary and Animal Sciences, Hisar (Haryana), India. Since, estrogens (female sex hormone) have neuroprotective effect (Kandi & Hayslett, 2011), therefore female mice were excluded from the present study. The animals were acclimatized at suitable laboratory conditions for seven days before the start of experiments and had free access to food and water. The experimental animals were approved by Institutional Animals Ethics Committee (Registration number 436) and care was taken as per the guidelines of Committee for the Purpose of Control and Supervision of Experiments on Animals, Ministry of Forests and Environment, India.

### Preparation of nanoparticles

Doxycycline hydrochloride loaded chitosan nanoparticles (DCNP) were prepared using modified ionotropic gelation method (Nagpal et al., 2013). Chitosan solution (0.05, 0.10 or 0.15% w/v) was prepared in 2% acetic acid solution and pH adjusted to 5.6 by the addition of sodium hydroxide solution. Then, doxycycline hydrochloride (40 mg) was added to the above solution followed by dropwise addition of aqueous solution of TPP (chitosan:TPP::3:1) with constant stirring at 1000 rpm for 60 min using magnetic stirrer. For coating, Tween 80 (0.5, 1 or 1.5%) was added dropwise followed by further stirring at 1000 rpm for 30 min. In order to separate DCNP, centrifugation (Remi Cool Equipments, Mumbai, India) was done at 15,000 rpm for 40 minute at 0 °C, which yielded nanoparticles in the form of pellet. Further, these pellets were washed and re-dispersed in 20 ml of double distilled water and sonicated using probe sonicator for 2 min. About 2 ml of this suspension was further diluted up to 10 times, sonicated for 2 minute and used for the analysis of particle size, zeta potential and size distribution. The undiluted DCNP suspension was lyophilized using lyophilizer (Alpha 2-4 LD Plus, CHRIST, Germany) after adding d-mannitol as a cryoprotectant in order to avoid particle agglomeration. Ranges for chitosan and Tween 80 for the optimization studies were set based on previous report (Nagpal et al., 2013) and optimization was done using two factor three level factorial design. Three levels each of the two factors factor *X*_1_ (i.e. percent chitosan concentration; w/v) and *X*_2_ (i.e. percent Tween 80 concentration; w/v), were adopted for as required by the design and the factor levels were suitably coded. Table 1 shows thirteen experimental runs performed using different levels of the selected factors. Blank nanoparticles (placebo) were prepared using the optimized values, but without the addition of doxycycline hydrochloride for the purpose of comparison.

**Table 1.** Composition, particle size and DEE of DCNP as per experimental design.

		Coded factor levels			
Formulation code	Trial number	*X*_1_	*X*_2_	Particle size (nm)	DEE (%)
7	1	−1	1	248	75.15
12	2	0	0	209	78.09
5	3	0	0	213	79.12
6	4	1	0	237	82.34
9	5	1	1	228	85.67
10	6	0	0	198	77.45
2	7	0	−1	243	74.56
8	8	0	1	218	76.16
11	9	0	0	202	76.19
4	10	−1	0	236	72.38
13	11	0	0	215	79.02
3	12	1	−1	257	86.62
1	13	−1	−1	229	72.78
Coded level	−1	0	+1		
*X*_1_: Chitosan (%)	0.05	0.15	0.25		
*X*_2_: Tween 80 (%)	1.0	1.5	2.0		

### Particle size analysis and zeta potential measurement

Particle size (hydrodynamic diameter) of the formulated DCNP was determined by dynamic light scattering technique, while zeta potential was measured using electrophoretic light scattering technique (ZetaSizer Nano ZS90; Malvern Instruments Limited, Malvern, UK).

### Drug entrapment efficiency (DEE)

The supernatant, after centrifugation of DCNP (15,000 rpm for 40 min), was collected, filtered through 0.22 μm membrane filter and amount of drug present was measured at 347 nm by UV–Visible spectrophotometer (Varian Cary 5000, Netherland). The amount of drug in supernatant was calculated using the equation *y* = 0.025*x* − 0.055, (*R*^2^ = 0.999), where *y* represents absorbance and *x* represents concentration (mg/ml). Amount of drug present in the supernatant was subtracted from the total amount of drug added and accordingly DEE was calculated.

### Optimization using 3^2^ factorial design

Design Expert Software, version 10.0.6.0 (Stat-Ease, Inc. Minneapolis, MN, USA) was employed to fit polynomial equations with attached interaction terms for the correlation of studied responses with chosen variables. Particles size and DEE were selected as response variables for systematic optimization. Optimized formulation was found by locating feasible space as well as exhaustive grid search was done for tracing the possible solution. Optimum solution was also provided by the software using the overlay plots. The optimized formulation (Tween 80 coated DCNP) was utilized for all the *in vitro* and *in vivo* studies.

### *In vitro* drug release

Equilibrium dialysis technique was employed for the study of *in vitro* drug release from optimized batch of DCNP in a USP Type II (Paddle type) apparatus. Optimized DCNP (equivalent to 50 mg doxycycline hydrochloride) were dispersed in 5 ml phosphate buffer saline (PBS) (pH 7.4) and placed in a dialysis membrane bag (molecular weight cut off 12,000–14,000, pore size 2.4 nm, Himedia, India), previously soaked in distilled water for 1 hour. The bag was placed in 300 ml PBS (37 ± 1 °C) at 50 rpm in dissolution apparatus. Aliquot of 5 ml was withdrawal at regular time intervals and replenished with equal volume of fresh dissolution media. The samples were analyzed, in triplicate, for the concentration of doxycycline hydrochloride using UV–Visible spectrophotometer (Varian Cary 5000, Netherland) at 347 nm against suitable blank and the amount of drug released at various time intervals was calculated. Mechanism of release was determined by fitting *in vitro* drug release data in different drug release kinetic models like zero order, first order, Hixson-Crowell, Higuchi and Korsemeyer Peppas.

### Transmission electron microscopy (TEM)

High resolution TEM (Tecnai G20; FEI Inc., Valley City, ND, USA) was used for the size and morphological characterization of the optimized DCNP batch. Formulated nanoparticles were suspended in HPLC grade water and sonicated (4 min) for disaggregation of nanoparticles. The sample was placed on a carbon coated grid (300 mesh), negatively stained with 2% phosphotungstic acid (pH 7.0) and analyzed using HR TEM at 200 kV.

### Fourier transform infrared (FTIR) spectroscopy

FTIR technique (Spectrum RX; Perkin Elmer, Waltham, MA, USA) was used to rule out any chemical interaction between chitosan, doxycycline hydrochloride and optimized DCNP (DCNP_opt_) by KBr pellet method.

### Animal study

The animals were divided into 14 groups and each group consisted of six animals. Group1 received 5% Tween 80 only, group 2 received ketamine (50 mg/kg, i.p.) only, group 3 received olanzapine (5 mg/kg, i.p.) followed by administration of ketamine (50 mg/kg, i.p.) after 30 minute of olanzapine, group 4 received placebo (50 mg/kg, p.o.) followed by administration of ketamine (50 mg/kg, i.p.) after 30 minute of placebo, group 5 received doxycycline hydrochloride (25 mg/kg, p.o.) followed by administration of ketamine (50 mg/kg, i.p.) after 30 min of doxycycline hydrochloride, group 6 received doxycycline hydrochloride (50 mg/kg, p.o.) followed by administration of ketamine (50 mg/kg, i.p.) after 30 min of doxycycline hydrochloride, group 7 received doxycycline hydrochloride (25 mg/kg, p.o.) followed by administration of ketamine (50 mg/kg, i.p.) after 30 minute of DCNP_opt_. Animals in groups 8–14 were treated as in groups 1–7, respectively. Animal in group 1–7 were used for biochemical estimations like AChE activity, GSH, MDA and TNF-α level, while animals in group 8–14 were used for biochemical estimations like GABA and dopamine levels in brain. Separate animals were used for histological studies.

### Behavioral assessments

Acute administration of ketamine produces hyperlocomotor activity, whereas on chronic administration, it produces depressive and cognitive symptoms of psychosis (Chatterjee et al., 2011). Behavioral assessments were carried out on 7th, 14th, 20th and 21st day of experiment in accordance to our previous study (Yadav et al., 2017). After behavioral assessments, blood was collected from retro-orbital site and then mice were sacrificed followed by isolation of their brain for biochemical studies.

#### Measurement of locomotor activity

Actophotometer was used to estimate the effect of DCNP_opt_ on locomotor activity. Each animal was placed in the activity chamber of the apparatus and total locomotor counts are expressed in terms of photo beam interruption during a time period of 10 min (da Silva et al., 2010).

#### Measurement of immobility duration

Forced swimming test (FST) was used to measure the effect of DCNP_opt_ on immobility period. In this test, animal was forced to swim in the glass chamber (25 × 15 × 25 cm) containing water up to the height of 15 cm (23 ± 2 °C). Acquisition of the state of immobility with minimum floating is considered as behavioral despair akin to depressive symptoms of psychosis. Mouse was placed for 2 min in the chamber for habituation and afterwards immobility period was recorded for the next 4 min (Chillar and Dhingra, 2013).

#### Measurement of step down latency (SDL)

Passive avoidance test is commonly used to measure the SDL which is a parameter to check the memory of small animals. The model consists of a chamber (27 × 27 × 27 cm) whose one wall is made of plexiglas, three walls made of wood, besides a grid floor with wooden platform. A 15 W bulb was used to illuminate the chamber and 20 V AC electric shocks were given to floor during the experiment. Experiment was performed into two learning sessions followed by one memory session. During the first learning session, animal was placed on wooden platform and when it stepped down on grid floor with all its paws before 60 s, shocks were delivered for 15 s. Next learning session was performed after an interval of 90 min, in which animals were free from shock. SDL is defined as the time taken by the animal to step down with all its paws from the wooden platform to the grid floor. Memory session was performed on next day and the animal was observed for 300 s to evaluate the cognitive function of animals. Animals were not provided shocks during the memory session (Soni and Parle, 2017).

### Biochemical estimations

#### Collection of blood for the estimation of TNF-α level

After the evaluation of behavioral parameters, animals in groups 1–7 were used for serum TNF-α level estimation. Blood was removed from retro-orbital plexus site and serum was separated by centrifugation at 3000 rpm for 15 min. Serum TNF-α level was estimated using ELISA reader (Erba, Mannheim, Germany).

#### Dissection and homogenization

Animals in groups 1–7 were sacrificed using cervical decapitation and immediately brain was removed, washed, weighed and divided into two equal parts. Homogenate of one part was prepared in 0.1 M phosphate buffer (pH 7.4) and used for the estimation of GSH, MDA and total protein level. Other part of brain was homogenized in phosphate buffer (pH 8) and then centrifuged at 3000 rpm for 10 min. The supernatant so obtained was used for the estimation of AChE activity.

#### Measurement of GSH

Ellman method was used to estimate the GSH level in brain. 1 ml of the prepared homogenate was precipitated by the addition of 1 ml of 4% trichloroacetic acid (TCA) and centrifuged at 5000 rpm for 10 min. Then, 2 ml disodium hydrogen phosphate buffer (0.3 M, pH 8.4) and double distilled water (0.4 ml) were added into 0.5 ml of the obtained supernatant. 0.25 ml of 0.001 M DTNB [5,5-dithiobis-(2-nitrobenzoic acid) dissolved in 1% w/v sodium citrate] was added into above mixture, followed by incubation at room temperature for 10 min. Developed yellow color was read at 412 nm using UV–Visible spectrophotometer. GSH level was calculated using molar extinction coefficient of 1.36 × 10^4^ M^−1 ^cm^−1^ and results expressed as µmole/mg brain protein (Ellman, 1959).

#### Measurement of MDA level

MDA level (a product of lipid peroxidation) was estimated in the form of thiobarbituric acid reactive substances (TBARS). About 0.5 ml brain homogenate and 0.5 ml tris–HCl buffer (pH 7.4) was mixed and incubated at 37 °C for 2 h. After incubation, 1 ml of TCA was added and centrifuged at 1000 rpm for 10 min. About 1 ml of supernatant so obtained was added with 1 ml of 0.67% thiobarbituric acid (TBA) and kept on boiling water bath for 10 minute. After cooling, 1 ml double distilled water was added and absorbance was read at 532 nm. TBARS level was calculated using extinction coefficient of 1.56 × 10^5^ M^−1 ^cm^−1^ and results are expressed as nmole of MDA/mg brain protein (Wills, 1964).

#### Measurement of AChE activity

The reaction mixture consists of 0.4 ml separated supernatant, 0.1 ml DTNB [5,5-dithio-bis-(2-nitrobenzoic acid)] and 2.6 ml of 0.01 M sodium phosphate buffer (pH 8) and its absorbance was taken on UV–Visible Spectrophotometer at 412 nm. Further, 0.02 ml acetylthiocholine iodide solution added, stand for 15 min and absorbance was again taken at 412 nm. The change in absorbance was calculated and results expressed as mmol/minute/gram of brain weight (Ellman, 1959).

### Neurochemical estimations

#### Dissection and homogenization

Animals in groups 8–14 were sacrificed and brain was isolated, washed, weighed and divided into two equal parts. One part was homogenized in 3 ml of HCl-butanol (0.1 M HCl in butanol) for the estimation of dopamine level while another part was homogenized in 5 ml of 0.01 M HCl for the estimation of GABA level.

#### Measurement of dopamine level

Schlumpf et al. (1974) method was used to quantify the level of dopamine in brain. About 0.8 ml supernatant was withdrawn by centrifugation of brain homogenate (2000 rpm for 10 min) and added to 0.25 ml of 0.1 M HCl and 2 ml of heptane. The obtained mixture was shaken for 10 min and centrifuged for the separation of aqueous phase. 0.01 ml of sodium acetate buffer (pH 6.9) and 0.05 ml of 0.4 M EDTA were added into 0.02 ml of the separated aqueous phase. Then, 0.01 ml iodine solution (0.1 M in ethanol) was added for oxidation and 0.5 ml sodium thiosulfate (5 M in NaOH) was used to stop oxidation after 2 min. After a gap of 90 s, acetic acid solution (0.5 ml of 10 M) was added and heated at 100 °C for 6 min. The mixture was read at 330/375 nm using spectrofluorimeter, after cooling. In the end, the tissue values (fluorescence of tissue extract minus fluorescence of tissue blank) were accessed with reference to internal reagent standard (fluorescence of internal reagent standard minus fluorescence of internal reagent blank). For the preparation of tissue blank, the reagents of oxidation step are mixed in reverse order (sodium thiosulfate before iodine) keeping all other parameters unchanged. Internal reagent standard was prepared by adding 2.5 ml HCl-butanol (0.1 M HCl in butanol), 0.125 ml distilled water into 500 ng dopamine standard, while internal reagent blank was formed by the addition of 0.125 ml distilled water into 2.5 ml HCl-butanol (0.1 M HCl in butanol) (Schlumpf et al., 1974).

#### Measurement of GABA level

About 0.1 ml of brain homogenate was mixed with 0.2 ml 0.14 M ninhydrin solution (in 0.5 M carbonate bicarbonate buffer of pH 9.95) and heated on water bath at 60 °C for 30 min After cooling, 5 ml of copper tartrate reagent (0.16% sodium carbonate, 0.03% copper sulfate, and 0.0329% tartaric acid) was mixed and stand for 10 min followed by reading of absorbance at 377/455 nm using spectrofluorimeter (Lowe et al., 1958).

### Histological studies

Formalin (10% v/v) was used to preserve the isolated brains, which were fixed into paraffin. Paraffin sections were prepared and stained with hematoxylin and eosin for histopathological studies.

### Statistical analysis

All the data are observed as mean ± SEM (*n* = 6). The difference between the different groups were analyzed using analysis of variance (ANOVA) followed by Tukey’s test using GraphPad Instat. The difference more than *p* < .05 was consider as statistically significant.

## Results

### Optimization by 3^2^ factorial design

3^2^ factorial design was used to find out the optimum concentration of chitosan and Tween 80 based on particles size and DEE measurements. Thirteen formulations were prepared in accordance with the chosen design and the effect of two factors, i.e. amount of chitosan (A) and amount of Tween 80 (B) was investigated on the two response variables viz. particle size and DEE. Average particle size of all the batches ranges between 198 and 257 nm, while the DEE was found between 72.38 and 86.62%. The generated polynomial equations were used for the analysis of the responses and analysis of variance was done using the statistical parameters like sum of squares, degree of freedom, mean sum of squares and *F* value using the software Design Expert. The significance of the developed model was judged based on *p* value, *F* value, correlation coefficient (*R*^2^) and adjusted correlation coefficient (adj *R*^2^).

The polynomial equations obtained using regression analysis for the responses (particle size and DEE) are as follows:

(1) $$\text{Particle size}=+\text{21}0.0\text{3 }+\text{1}.\text{5}0\;A-\text{5}.\text{83 }B\;-\text{12}.00\;AB+\text{19}.\text{88 }A^{\text{2}}+\text{13}.\text{88 }B^{\text{2}}$$

(2) DEE=+78.12+5.72 A+0.51 B

In the above regression equations, the positive and negative symbols before the coefficients (main and interaction terms) represent their synergistic or antagonistic effect on the responses, respectively. The significance of the developed models was established on the basis of the calculated *F*-value and *p*-value; in our case, it is less than .05 at 95% confidence level. The values for particle size are *Df* = 5, *F* = 7.91, *p* = .0085, *R*^2^ = 0.8496, Adjusted *R*^2^ = 0.7421, while for DEE *Df* = 2, *F* = 23.60, *p* = .0002, *R*^2^ = 0.8252, Adjusted *R*^2^ = 0.7902. Significance of the developed model is further represented by sufficiently high values of *R*^2^ and adjusted *R*^2^. The large *F*-value obtained with Y3, indicates that the effect variance is much higher than the error variance.

Using the desirability approach for numerical optimization and desired responses, solution for optimized batch was obtained by setting the parameters viz. minimum particle size (198–250 nm) with maximum entrapment efficiency (70–90%). More importance was given to particle size than DEE. The parameters as suggested by Design Expert software for optimum formulation were chitosan 0.4765 (coded value) and Tween 80 0.4212 (coded value) which resulted in the formulation of nanoparticles with a particle size of 212.86 nm and DEE 80.449%. The percent mean error between predicted values and experimental values was found to be 9.69% (for particle size) and 2.84% (for DEE) indicating the success of optimization process for DCNP.

**Figure 1.**
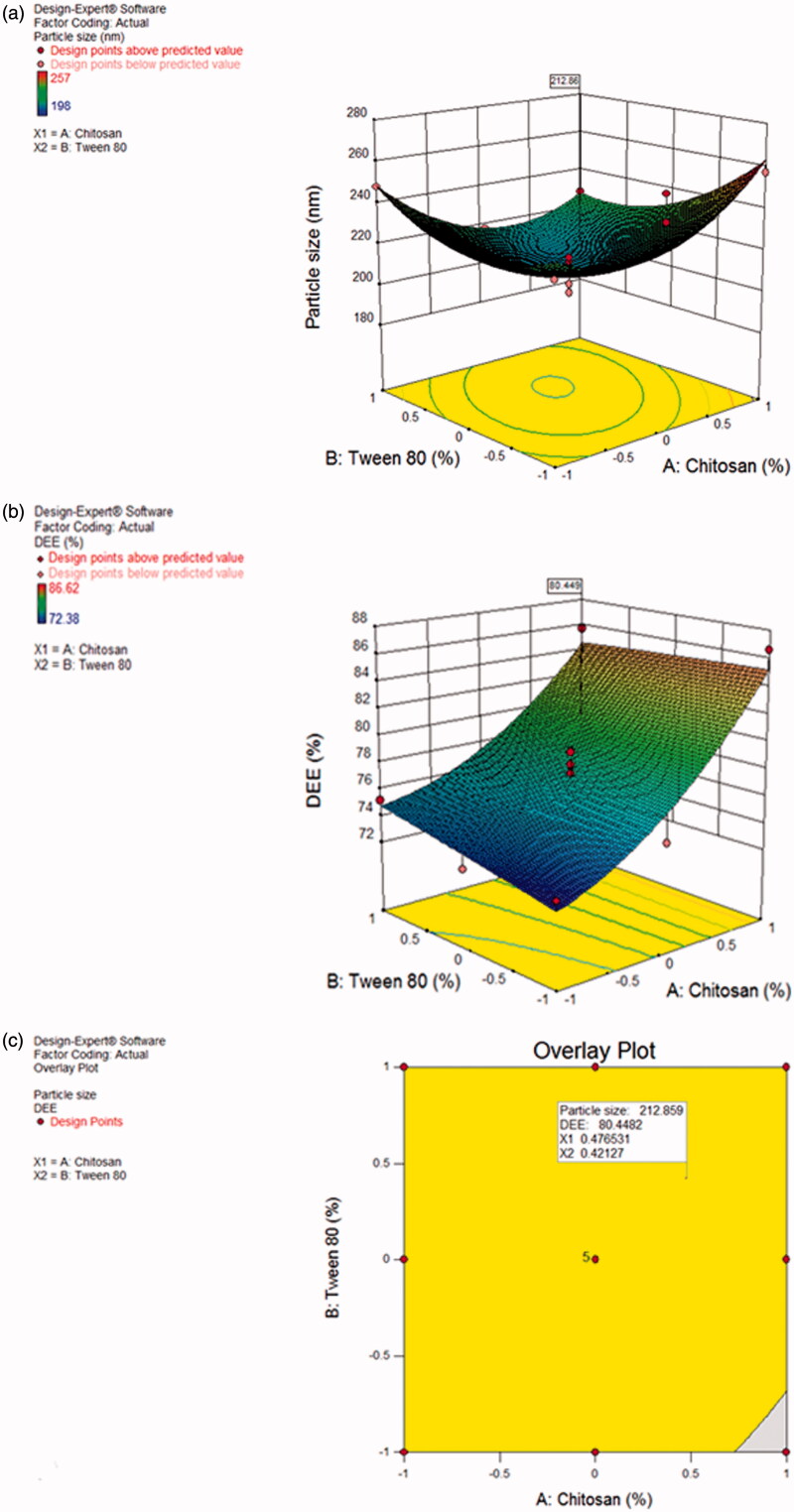
(a) 3D response surface graph of DCNP showing the effect of chitosan and Tween 80 on particle size. (b) 3 D response surface graph of DCNP showing the effect of chitosan and Tween 80 on DEE. (c) Overlay plot showing the location of optimized batch of DCNP.

The 3D surface plots showing the effect of formulation factors on the response variables, have been depicted in Figure 1(a). Both the chosen factors were found to affect significantly the response variables. As the concentration of chitosan was increased from 0.05% to 0.15%, particle size increased with a minimum in between. Similar effect was observed by increasing the concentration of Tween 80 from 0.5% to 1.5%. On increasing the concentration of Tween 80, particle size first decreases and then increases (Figure 1(a)). Chitosan and Tween 80 in their squared terms are contributing more towards increase in particle size, as shown by their coefficients in Equation (1), although the alone effect of Tween 80 as well as its interaction term with chitosan results in decrease of particle size. Figure 1(b) shows the effect of chitosan and Tween 80 on the DEE. Although, increase in the concentration of both chitosan and Tween 80 leads to increase in DEE, but the effect of chitosan is more pronounced as compared to Tween 80. This fact is also shown by higher coefficient of chitosan than Tween 80 in Equation (2). Increase in DEE with increase in polymer concentration may be attributed to better network formation by the polymer. Figure 1(c) also shows the predicted parameters for the optimized batch (particle size 212.86 nm and DEE 80.449%).

### Transmission electron microscopy (TEM)

**Figure 2.**
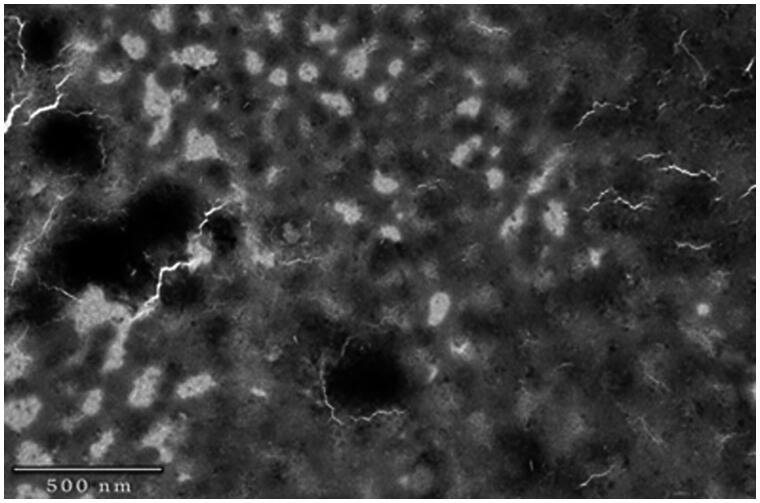
TEM image of DCNP_opt_.

TEM analysis of DCNP_opt_, shown in Figure 2, indicates that the particles were semi-spherical and spherical in shape. Particle size observed by TEM was lower than that observed by particle size analyzer as zetasizer measures the apparent diameter, which includes hydrodynamic layers around the nanoparticles.

### Fourier transform infrared (FTIR) spectroscopy

**Figure 3.**
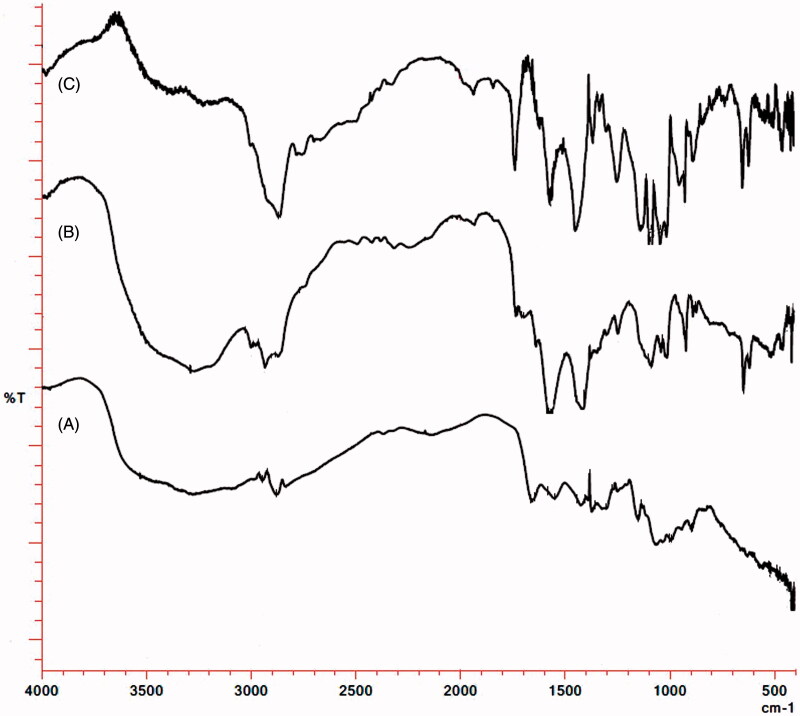
FTIR spectra of (A) Chitosan (B) Doxycycline hydrochloride (C) DCNP_opt_.

FTIR spectra of pure doxycycline is shown in Figure 3, which is characterized by the presence of broad band in the region 3500–3100 cm^−1^ due to the O–H stretching vibrations. The presence of aromatic ring in the structure of doxycycline hydrochloride is shown by aromatic C–H stretching at 3001 cm^−1^ and strong absorption band at 923 cm^−1^ due to out of plane bending of ring C–H bonds. The band at 1702 cm^−1^ is due to presence of carbonyl group (C = O stretching), while the C–C–C stretching and C–C(=O)–C bending is shown at 1248 cm^−1^. Amide I band due to carbonyl absorption at 1645 cm^−1^ and amide II band due to N–H bending at 1572 cm^−1^ confirms presence of amide group in the structure of doxycycline hydrochloride, however, N–H stretching bands overlaps with the O–H stretching bands in the region 3500–3100 cm^−1^. Strong absorption at 1093 cm^−1^ is attributed to C–N stretching of amine group. On the other hand, FTIR spectra of chitosan shows intense broad band in the region 3600–3150 cm^−1^ due to O–H stretching vibrations. The C–H stetching vibrations are present at 2925 cm^−1^ and N–H stretching vibrations merges with O–H stretching vibrations in the region 3600–3150 cm^−1^. N–H bending (scissoring) of amine is present at 1645 cm^−1^, while C–N stretching is present at 1078 cm^−1^. Peak of C–O–C in the glucopyranose ring is present at 1030 cm^−1^, while the specific bands of β (1 → 4) glycosidic bridge at 1154 and 898 cm^−1^ (Figure 3). The FTIR spectra corresponding to DCNP_opt_ ruled out any chemical interaction between drug and polymer during the formulation as their individual characteristic bands are present in the spectra of the formulation DCNP_opt_.

### *In vitro* drug release study

More than 95% drug release was obtained over a time span of 24 h from DCNP_opt_ batch showing sustained release pattern of the drug from the formulation. In the initial 2 h, about 36% drug was released, thereby showing the drug release from the start of dissolution. Fitting of drug release data in various release kinetic equations (mathematical models) resulted in correlation coefficients (*R*^2^) 0.854, 0.593, 0.999, 0.962, and 0.691 for zero order, first order, Higuchi, Korsemeyer-Peppas and Hixson Crowell models, respectively. Higuchi model was depicted as best fit model with *R*^2^ value of 0.999, which represents drug release through matrix system. Here drug release is as a diffusion process based on Fick’s law (Costa & Lobo, 2001). So it was concluded that release of doxycline from DCNP_opt_ followed diffusion controlled release. Initial release of drug may be ascribed to faster migration of drug molecules from the vicinity of the DCNP_opt_ surface and thereafter, sluggish release of the drug may be due to solvation of chitosan in dissolution fluid as well as diffusion of the entrapped drug through swollen polymer matrix combined with erosion at boundary layer of matrix system.

### Behavioral assessments

#### Locomotor activity

As compared to vehicle, ketamine (50 mg/kg, i.p.) significantly increased locomotor activity after administration for seven days. Olanzapine (5 mg/kg, i.p.) significantly (*p* < .001) decreased the hyperlocomotor activity induced by ketamine. Pure doxycycline hydrochloride (25 and 50 mg/kg, p.o.) (*p* < .001) and DCNP_opt_ (25 mg/kg, p.o.) (*p* < .001), significantly [*F* (6, 35) = 31.412] decreased the ketamine induced hyperlocomotor activity. Furthermore, DCNP_opt_ (25 mg/kg, p.o.) was also remarkable to diminish the locomotor counts in comparison to pure doxycycline hydrochloride even at 50 mg/kg, p.o. The effect of DCNP_opt_ (25 mg/kg, p.o.) on locomotor activity was statistically equivalent to olanzapine (5 mg/kg, i.p.) (Table 2).

**Table 2.** Effect of doxycycline hydrochloride and DCNP_opt_ on various behavioral and biochemical parameters.

	Vehicle	Keta (50 mg/kg)	Keta + Olz (5 mg/kg)	Keta + Placebo (50 mg/kg)	Keta + Doxy (25 mg/kg)	Keta + Doxy (50 mg/kg)	Keta + DCNP_opt_ (25 mg/kg)
Locomotor counts	283.33 ± 9.61	502.66 ± 7.46*	359.33 ± 10.44^a^	490.50 ± 11.55	444.83 ± 19.61	394.16 ± 8.42^a^	304.33 ± 23.4^a^^,^^x^^,^^q^
Stereotyped behaviors	2.50 ± 0.34	27.66 ± 1.14*	7.83 ± 0.6^a^	26.50 ± 0.84	21.16 ± 1.74**^**b**^**	18.66 ± 1.47^a^	11.16 ± 1.49^a^^,^^x^^,^^q^
Immobility duration (s)	72.5 ± 1.61	149.83 ± 1.88*	81.83 ± 1.49^a^	146.33 ± 1.99	125.16 ± 2.93^a^	114.50 ± 3.08^a^	81.16 ± 5.16^a^^,^^x^^,^^p^
SDL (s)	237.66 ± 14.55	76.83 ± 3.98*	186 ± 2.22^a^	74.16 ± 4.01	80.16 ± 4.52	80.50 ± 4.52	90.83 ± 3.17^#^
TNF-α (ng/ml)	125.16 ± 3.65	499.66 ± 9.62*	128.00 ± 2.58^a^	495.33 ± 12.02	378.66 ± 5.56^a^	330.33 ± 15.51^a^	147.33 ± 4.77^a^^,^^x^^,^^p^
GSH (nmol/mg protein)	0.0051 ± 0.00020	0.0015 ± 0.00011*	0.0051 ± 0.00016^a^	0.0016 ± 9.38E-50	0.0029 ± 2.72E-04^a^	0.0038 ± 0.00011^a^	0.0053 ± 1.69E-03^a^^,^^x^^,^^p^
MDA (nmol/mg protein)	0.252 ± 0.014	0.563 ± 0.019*	0.228 ± 0.011^a^	0.558 ± 0.020	0.424 ± 0.010^a^	0.396 ± 0.007^a^	0.208 ± 0.08^a^^,^^x^^,^^p^
AchE (nmol/min/g protein)	38.82 ± 1.24	65.24 ± 2.05*	36.68 ± 1.92^a^	64.79 ± 3.90	64.74 ± 1.68	64.25 ± 3.11	62.67 ± 1.41^#^
Dopamine (pg/mg brain)	427.38 ± 27.11	981.73 ± 30.68*	429.62 ± 29.03^a^	978.40 ± 25.40	688.33 ± 21.12^a^	642.93 ± 27.21^a^	435.16 ± 21.55^a^^,^^x^^,^^p^
GABA (μg/g brain)	192.58 ± 6.75	107.80 ± 6.06*	208.88 ± 8.80^a^	106.42 ± 6.11	147.50 ± 3.22^a^	155.99 ± 3.56^a^	211.30 ± 5.62^a^^,^^x^^,^^p^

Values are expressed as mean ± SEM (*n* = 6).

* *p* < .001 vs vehicle group.

^a^ *p* < .001 vs ketamine group.

^x^ *p* < .001 vs Keta + Doxy (25 mg/kg).

^p^ *p* < .001 vs Keta + Doxy (50 mg/kg).

^q^ *p* < .01 vs Keta + Doxy (50 mg/kg).

^#^ *p* < .001 vs olanzapine.

Keta: Ketamine; Olz: Olanzapine; Doxy: Doxycycline hydrochloride; DCNP_opt_: Optimized Doxycycline hydrochloride Chitosan Nanoparticles.

#### Stereotyped behaviors

Ketamine (50 mg/kg, i.p.) administration for 10 successive days induced stereotyped behaviors in mice. Olanzapine (5 mg/kg, i.p.) significantly (*p* < .001) decreased the stereotyped behaviors induced by ketamine. Treatment with pure doxycycline hydrochloride (25 and 50 mg/kg, p.o.) (*p* < .05 and *p* < .001, respectively) and DCNP_opt_ (25 mg/kg, p.o.) (*p* < .001) was significant [*F* (6, 35) = 64.646] to decrease the ketamine produced stereotyped behaviors. Moreover, DCNP_opt_ (25 mg/kg, p.o.) was also found to be remarkable to lessen the stereotyped behaviors as compared to pure doxycycline hydrochloride at the dose level of 50 mg/kg, p.o. The effect of DCNP_opt_ (25 mg/kg, p.o.) on stereotyped behaviors was not significant in comparison to olanzapine (5 mg/kg, i.p.) which reveals effect of DCNP_opt_ is statistically equivalent to olanzapine for the treatment of ketamine induced stereotyped behaviors (Table 2).

#### Immobility duration

Immobility duration in FST was significantly increased with the administration of ketamine (50 mg/kg, i.p.) for 14 successive days, which was effectively decreased by the treatment of pure doxycycline hydrochloride (25 and 50 mg/kg, p.o.) (*p* < .001) and DCNP_opt_ (25 mg/kg, p.o.) (*p* < .001) [*F* (6, 35) = 126.09]. However, DCNP_opt_ even at the dose of 25 mg/kg was more effective than pure drug at the dose of 50 mg/kg in alleviating the immobility duration in FST. Olanzapine (5 mg/kg, i.p.) significantly (*p* < .001) decreased the immobility duration increased by ketamine. The effect of DCNP_opt_ (25 mg/kg, p.o.) on immobility duration was statistically equivalent to olanzapine (5 mg/kg, i.p.) (Table 2).

#### Step-down latency

Significant decrease in step-down latency in passive avoidance test has been observed with the administration of ketamine (50 mg/kg, i.p.) for 20 successive days, which indicates memory loss. Pure doxycycline hydrochloride (25 and 50 mg/kg, p.o.) and DCNP_opt_ (25 mg/kg, p.o.) did not show any significant difference in step down latency in comparison to ketamine. Olanzapine (5 mg/kg, i.p.) significantly (*p* < .001) [*F*= (6,35) = 101.82] increased the step-down latency decreased by ketamine. However, DCNP_opt_ (25 mg/kg, p.o.) did not produced increase in step-down latency in comparison to olanzapine (5 mg/kg, i.p.) (*p* < .001) (Table 2).

### Biochemical estimations

#### Measurement of TNF-α level

Serum TNF-α level was increased with ketamine (50 mg/kg, i.p.) administration for 21 successive days. Treatment with pure doxycycline hydrochloride (25 and 50 mg/kg, p.o.) and DCNP_opt_ (25 mg/kg, p.o.) significantly [*p* < .001, *F* (6, 35) = 270.75] decreased TNF-α level as compared to ketamine treated animals. DCNP_opt_ (25 mg/kg, p.o.) was also remarkable to reduce TNF-α level as compared to pure doxycycline hydrochloride (25 and 50 mg/kg, p.o.) indicating better penetration into systemic circulation. Olanzapine (5 mg/kg, i.p.) significantly (*p* < .001) decreased the serum TNF-α level increased by ketamine. Also, the effect of DCNP_opt_ (25 mg/kg, p.o.) on serum TNF-α level was statistically equivalent to olanzapine (5 mg/kg, i.p.) (Table 2).

#### Measurement of GSH level

Brain GSH level was decreased by the administration of ketamine (50 mg/kg, i.p.) for 21 successive days, which was significantly increased with the treatment of pure doxycycline hydrochloride (25 and 50 mg/kg, p.o.) and DCNP_opt_ (25 mg/kg, p.o.) [*p* < .001, *F* (6, 35) = 90.850]. DCNP_opt_ was found to be more effective to increase GSH level than pure drug as DCNP_opt_ showed pronounced brain targeted effect at the dose of 25 mg/kg than the pure drug at the dose of 50 mg/kg. Olanzapine (5 mg/kg, i.p.) significantly (*p* < .001) increased the brain GSH level decreased by ketamine. The effect of DCNP_opt_ (25 mg/kg, p.o.) on brain GSH level was found to be statistically equivalent to olanzapine (5 mg/kg, i.p.) (Table 2).

#### Measurement of MDA level

Lipid peroxidation was increased with the administration of ketamine (50 mg/kg, i.p.) for 21 successive days as indicated by increased MDA level in brain. However, treatment with pure doxycycline hydrochloride (25 and 50 mg/kg, p.o.) and DCNP_opt_ (25 mg/kg, p.o.) was significant [*p* < .001, *F* (6, 35) = 116.93] to decrease MDA level in mice brain as compared to ketamine treated animals. Brain targeting efficiency of DCNP_opt_ is further illustrated by remarkable decrease in MDA level at a dose of 25 mg/kg, which was even lower than pure doxycycline hydrochloride at the dose of 50 mg/kg. Olanzapine (5 mg/kg, i.p.) significantly (*p* < .001) decreased the MDA level increased by ketamine. The effect of DCNP_opt_ (25 mg/kg, p.o.) on MDA level was found to be statistically equivalent to olanzapine (5 mg/kg, i.p.) (Table 2).

#### Measurement of AChE activity

On administration of ketamine (50 mg/kg, i.p.) for 21 successive days, brain AChE activity was increased. However, treatment with pure doxycycline hydrochloride (25 and 50 mg/kg, p.o.) and DCNP_opt_ (25 mg/kg, p.o.) for 21 successive days was not significant to reduce AChE activity as compared to ketamine. Olanzapine (5 mg/kg i.p.) significantly (*p* < .001) decreased the AChE activity increased by ketamine. However, the effect of DCNP_opt_ (25 mg/kg, p.o.) on AChE activity was statistically significant as compared to olanzapine (5 mg/kg, i.p.) (*p* < .001) (Table 2).

### Neurochemical estimations

#### Measurement of dopamine level

Brain dopamine level was increased with the administration of ketamine (50 mg/kg, i.p.) for 21 successive days, which was significantly reversed by treatment with pure doxycycline hydrochloride (25 and 50 mg/kg, p.o.) and DCNP_opt_ (25 mg/kg, p.o.) [*p* < .001, *F* (6, 35) = 172.89]. In addition, DCNP_opt_ (25 mg/kg, p.o.) significantly decreased dopamine level as compared to pure doxycycline hydrochloride (25 and 50 mg/kg, p.o.) showing added bioavailability in brain. Olanzapine (5 mg/kg, i.p.) significantly (*p* < .001) decreased the brain dopamine level increased by ketamine. The effect of DCNP_opt_ (25 mg/kg, p.o.) on brain dopamine level was statistically equivalent to olanzapine (5 mg/kg, i.p.) (Table 2).

#### Measurement of GABA level

The level of GABA in brain was decreased with the administration of ketamine (50 mg/kg, i.p.) for 21 successive days. Olanzapine (5 mg/kg, i.p.) significantly (*p* < .001) increased the GABA level decreased by ketamine. Also, treatment with pure doxycycline hydrochloride (25 and 50 mg/kg, p.o.) and DCNP_opt_ (25 mg/kg, p.o.) significantly [*p* < .001, *F* (6, 35) = 54.826] increased GABA level in mice brain as compared to ketamine treated animals. Additionally, DCNP_opt_ (25 mg/kg) was also significant to increase GABA level as compared to pure doxycycline hydrochloride (even at 50 mg/kg), indicating improved bioavailability and penetration in brain through blood brain barrier after coating of nanoparticulate doxycycline hydrochloride with Tween 80. The effect of DCNP_opt_ (25 mg/kg, p.o.) on GABA level was statistically equivalent to olanzapine (5 mg/kg, i.p.) (Table 2).

### Histopathological studies

**Figure 4.**
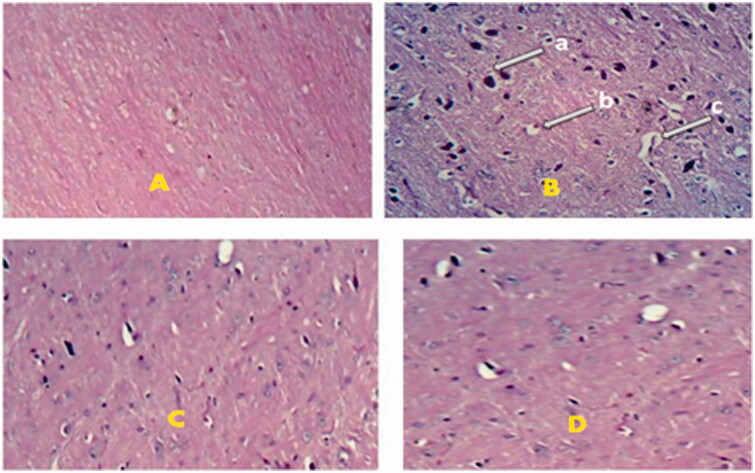
Effect of DCNP_opt_ on histopathological changes of brain. A = Vehicle treated brain, B = Ketamine treated brain, C = Doxycycline hydrochloride treated brain, D = DCNP_opt_ treated brain. ‘a’ – hyperchromatic nuclei, ‘b’ – perinuclear vacuolization, ‘c’ – dilated vascular channels.

Above findings were also confirmed by histopathological examinations. Chronic treatment with DCNP_opt_ (25 mg/kg, p.o.) for 21 successive days was significant to reverse ketamine (50 mg/kg, i.p.) produced histopathlogical alterations such as perinuclear vacuolization, dilated vascular channels and hyperchromatic nuclei in brain as shown in Figure 4.

## Discussion

The present study was designed to develop novel, optimized, biodegradable, biocompatible, nontoxic and economical brain targeting drug delivery system consisting of Tween 80 coated doxycycline hydrochloride nanoparticles and studied against ketamine induced psychosis in animal models for their possible behavioral, biochemical, neurochemical and histological alterations. Being reported as a stable, nontoxic, biodegradable, biocompatible and inexpensive biopolymer potentiating brain uptake of several drugs for the management of psychiatric disorders (Mandala Rayabandla et al., 2010; Sarısozen et al., 2010; Hansraj et al., 2015), chitosan was selected for the present study. In order to target the brain, coating of nanoparticles was done with Tween 80, a non-ionic surfactant, owing to its brain specificity by binding with plasma Apo-E lipoprotein that mimics low density lipoproteins (LDL), which further attach with LDL receptors on brain micro vascular endothelial cells (Kreuter, 2001). Moreover, particle size plays very important role in controlling the rate of endocytosis across the blood brain barrier (Nagpal et al., 2013). Particle size of all the thirteen formulated batch of DCNP batches was below 300 nm, while the size of nanoparticles for optimized batch of DCNP was below 240 nm as required for brain targeting. The size of nanoparticles was found to decrease with increasing amount of Tween 80 possibly due to formation of smaller droplets during the formulation of nanoparticles. TEM analysis of DCNP_opt_ batch revealed their sub-spherical and spherical shape. The particle size of DCNP_opt_ obtained from TEM imaging was lesser than zetasizer, as zetasizer measures hydrodynamic volume, which is not the case with TEM. The average poly dispersity index was below 1.0 showing monodisperse, homogenous particle size distribution. Further, zeta potential value of DCNP_opt_ was highly positive indicating good physical stability, which may be due to the unreacted amino acid residual on chitosan backbone. DEE of optimized formulation was 78.16%, which was almost closer to the predicted value. Increasing the concentration of chitosan leads to increase in DEE which may be attributed to better polymer network formation and lesser leaching of the drug. Fitting of different kinetics models indicated that drug release from nanoparticles follows Higuchi model (*R*^2^ value 0.999) represents drug release through matrix system based on the principle of diffusion, hence, the release of doxycline from DCNP followed diffusion controlled release.

In order to evaluate the effect of the DCNP_opt_ against ketamine induced psychosis, *in vivo* animal models were used. Several evidences suggest the role of NMDA receptor antagonists in the development of positive, negative and cognitive symptoms of psychosis (Yadav et al., 2017). Ketamine, a NMDA receptor antagonist, has been reported to produce behavioral, biochemical and neurochemical alterations in rodents similar to human psychosis (Chatterjee et al., 2012). Neuropharmacologically, it has been studied that ketamine antagonizes the NMDA receptors located on GABAergic neurons resulting in increased dopamine level in prefrontal cortex region of the brain leading to stimulate locomotor activity and stereotyped behaviors, representing positive symptoms of psychosis (Chatterjee et al., 2011; Yadav et al., 2017). In the present study, ketamine significantly induced positive symptoms of psychosis in mice as indicated by increased locomotor counts and stereotypic behaviors. DCNP_opt_ was significant to attenuate locomotor counts and stereotypic behaviors as compared to ketamine as well as pure doxycycline hydrochloride treated animals even at much lower dose representing successful penetration in brain. These effects were confirmed by neurochemical estimations; DCNP_opt_ augmented the level of GABA and diminished the level of dopamine than pure doxycycline hydrochloride as well as ketamine even at much lower doses again showing more permeability through blood brain barrier. Our observations are in accordance with previous studies (Corti et al., 1999; Kellendonk et al., 2006), where doxycycline hydrochloride attenuated dopamine receptors gene expression eventually decreasing dopamine concentration; this report is further strengthened by our present study.

Reactive oxidative species (ROS) and hyperactivated microglial cell are related with negative symptoms of psychosis along with other psychiatric disorders. Ketamine is also connected with oxidative damage by producing ROS and increasing the release of inflammatory cytokines (TNF-α) by the activation of microglial cells (Zugno et al., 2014; Yadav et al., 2017). In the present investigation, Ketamine was found to induce negative symptoms of psychosis as evidenced by increased immobility duration in FST. DCNP_opt_ effectively decreased immobility duration at much lower doses as compared to ketamine and pure doxycycline hydrochloride treated mice, demonstrating the increased penetration into brain by coating of nanoparticles with Tween 80. The potent pro-inflammatory cytokine (TNF-α) promote inflammatory signaling. An increase in free radicals causes overproduction of MDA, which is the final product of polyunsaturated fatty acids peroxidation in the cells. Reduced GSH molecule neutralizes free radicals by bonding through its extra electron to the ROS molecules capturing free radical electron. In biochemical studies, DCNP_opt_ was significant to diminish the level of TNF-α and lipid peroxidation, while increased GSH level as compared to pure doxycycline hydrochloride and ketamine resulting in improved anti-inflammatory and antioxidant effects; thus, better pharmacodynamic effects due to improved bioavailability in brain. Moreover, available literature on anti-inflammatory (Tilakaratne & Soory, 2014; Omolu et al., 2017) and antioxidant (Antonio et al., 2014) effects of doxycycline hydrochloride supports our study. These observations collectively suggest the protective effect of DNCP_opt_ against depressive symptoms of psychosis at lower dose.

Acetylcholine is a one of the main neurotransmitter involved in formation of memory, which is degraded by AChE enzyme (Soni & Parle, 2017). Ketamine has already been established to reduce the concentration of acetylcholine in hippocampal region of brain by the blockade of nAChR with increase of AChE activity related to cause cognitive symptoms of psychosis (Chatterjee et al., 2012). Our study also demonstrated that ketamine was found to produce cognitive symptom as indicated by decrease the time of SDL in passive avoidance test. DCNP_opt_ and pure doxycycline hydrochloride treatment showed non-significant effect revealed by behavioral and AChE activity assessments.

Furthermore, administration of DCNP_opt_ significantly decreased ketamine produced histopathlogical changes like perinuclear vacuolization, dilated vascular channels and hyperchromatic nuclei in brain, demonstrating the efficacy of DCNP_opt_ at much lower dose than pure drug. These observations again support the enhanced penetration of doxycycline hydrochloride through blood brain barrier when given in the form of Tween 80 coated nanoparticles. The results of DCNP_opt_ against positive and negative symptoms of psychosis are comparable to olanzapine, an established antipsychotic agent used clinically, which has been taken as a standard drug in the present study. Collectively, behavioral, biochemical and histopathlogical results showed pure doxycycline hydrochloride as well as its nanoformulations were able to diminish the ketamine induced positive and negative symptoms of psychosis; the effects produced by DCNP_opt_ at dose of 25 mg/kg are even better than the effects produced by pure doxycycline hydrochloride even at 50 mg/kg dose, showing significant reduction in dose by its brain targeting through nanoparticulate formulation.

## Conclusion

The present study suggested a novel nanoparticulate formulation of doxycycline hydrochloride for the treatment of ketamine induced psychotic symptoms in animal models. The optimization was performed to evaluate the effect of concentration of chitosan and Tween 80 on DEE and particle size. The observed parameters for DCNP_opt_ were found to be significant as compared to software predicted values. The *in vitro* drug release was found to be 95.44% in 24 h, indicating controlled release of doxycycline hydrochloride from DCNP_opt_. Doxycycline hydrochloride has showed noticeable antipsychotic activity, otherwise used for its antibiotic activity; increased GABA and GSH level and decreased MDA, TNF-α and dopamine level could probably be involved fort its protective mechanism in the treatment of ketamine induced psychotic symptoms. Observed antipsychotic effects of doxycycline hydrochloride in the form of DCNP_opt_ at significantly lower dose than its pure form indicates better brain uptake of doxycycline hydrochloride through blood brain barrier achieved through brain targeting by Tween 80 coating of nanoparticles. This indicates the effectiveness of nanoparticulate drug delivery system in targeting of hydrophilic drugs by incorporating in Tween 80 coated chitosan nanoparticles for the potential treatment of neuropsychiatric disorders.
